# Dysautonomia in Children with Post-Acute Sequelae of Coronavirus 2019 Disease and/or Vaccination

**DOI:** 10.3390/vaccines10101686

**Published:** 2022-10-09

**Authors:** Reiner Buchhorn

**Affiliations:** Praxis für Kinder- und Jugendmedizin, Kinderkardiologie und Erwachsene mit Angeborenen Herzfehlern, Am Bahnhof 1, 74670 Forchtenberg, Germany; buchrein@gmail.com

**Keywords:** SARS-CoV-2 virus, COVID-19, dysautonomia, heart rate variability, vaccination, long COVID

## Abstract

Long-term health problems such as fatigue, palpitations, syncope, and dizziness are well-known in patients after COVID-19 (post-acute sequelae of coronavirus (PASC)). More recently, comparable problems have been noticed after the SARS-CoV-2 vaccination (post-VAC). The pathophysiology of these problems is not well-understood. Methods: In 38 children and young adults, we tested if these health problems were related to dysautonomia in an active standing test (Group 1: 19 patients after COVID-19; Group 2: 12 patients with a breakthrough infection despite a vaccination; and Group 3: 7 patients after a vaccination without COVID-19). The data were compared with a control group of 47 healthy age-matched patients, as recently published. Results: All patients had a normal left ventricular function as measured by echocardiography. Significantly elevated diastolic blood pressure in all patient groups indicated a regulatory cardiovascular problem. Compared with the healthy control group, the patient groups showed significantly elevated heart rates whilst lying and standing, with significantly higher heart rate increases. The stress index was significantly enhanced in all patient groups whilst lying and standing. Significantly decreased pNN20 values, mostly whilst standing, indicated a lower vagus activity in all patient groups. The respiratory rates were significantly elevated in Groups 1 and 2. Conclusion: The uniform increase in the heart rates and stress indices, together with low pNN20 values, indicated dysautonomia in children with health problems after COVID-19 disease and/or vaccination. A total of 8 patients fulfilled the criteria of postural orthostatic tachycardia syndrome and 9 patients of an inappropriate sinus tachycardia, who were successfully treated with omega-3 fatty acid supplementation and pharmacotherapy.

## 1. Introduction

Coronavirus disease 2019 (COVID-19) is caused by a SARS-CoV-2 virus infection. After the acute disease, ongoing health problems of COVID-19 are now being identified with the most common being fatigue, shortness of breath, chest pain, and palpitations that may indicate cardiovascular disease. Furthermore, patients can suffer from difficulty concentrating, arthralgia, low-grade fever, and headaches that may indicate ongoing inflammation. These ongoing health problems have been termed Post-Acute Sequelae of Coronavirus 2019 Disease (PASC) [1]. However, as most symptoms of PASC are not clearly defined, the number of affected patients cannot be clearly stated. However, PASC is an increasing health problem in adults and children. Recently, among SARS-CoV-2-positive children, the most common long-term symptom was fatigue or weakness (1.1%) [2]. 

Additionally, the impact of the SARS-CoV-2 vaccination on PASC is controversial. Most physicians speculate that the vaccination may protect against long-term health problems [3] and can even be used for therapy [4]. Recent findings suggest that a vaccination before an infection confers only a partial protection against PASC. In addition, new data have been published that show that the SARS-CoV-2 vaccination may cause PASC [5].

Dysautonomia has been found in patients who suffer from PASC [6]. Moreover, dysautonomia may be linked to long-term health problems after COVID-19 as well as the SARS-CoV-2 vaccination [7]. Currently, there are no established therapies for PASC and the concept of “dysautonomia” could open up therapeutic approaches to which there is already a guideline recommendation [8].

We now introduce our data of 38 children who suffered from PASC after COVID-19 (N = 19), a breakthrough infection after a vaccination (N = 12), or PASC-like long-term health problems after the SARS-CoV-2 vaccination (N = 7).

## 2. Methods

The study was a retrospective analysis of 38 consecutive children and adolescents who had an active standing test of the cause of ongoing health problems for more than 11 weeks on average after COVID-19 disease or vaccination. In Group 1, there were 19 patients after COVID-19, Group 2 contained 12 patients with a breakthrough SARS-CoV-2 infection despite a vaccination, and Group 3 had 7 patients after a vaccination without COVID-19. After excluding other clearly defined diseases, we did not assess the subjective clinical problems and included all patients who displayed ongoing health problems after COVID-19 and/or SARS-CoV-2 vaccination. The most common symptoms were fatigue or weakness as well as palpitations, chest pain, dizziness, shortness of breath, inattention, and gastrointestinal problems. COVID-19 disease was proven by a PCR in all patients. All patients had a complete cardiovascular check-up with ECG, a fully automatic photoplethysmography blood pressure test, and echocardiography. 

The healthy control group included 47 healthy adolescents (mean age = 14.2 ± 3.8 years) with a normal body mass index and without any somatic or psychosomatic diseases. These data were collected prior to the pandemic. The groups were stratified according to their age, height, weight, and gender (Table 1).

### 2.1. Active Standing Test

All patients had at least one active standing test with a continuous measurement of their heart rate during 5 min in a supine position and 5 min during active standing using an HRV Scanner™ (BioSign GmbH, Ottenhofen, Germany). We performed the blood pressure tests in the supine position. 

### 2.2. HRV

The measurement and interpretation of the HRV parameters in the current sample were standardised according to the task force guidelines.

- A short-time HRV analysis was performed using the HRV Scanner™ (BioSign GmbH, Ottenhofen, Germany) at two time periods: (1) in a supine position; and (2) 5 min of active standing. The HRV measurements were based on the RR intervals of normal QRS complexes (NN intervals) of the two 5 min intervals.

#### 2.2.1. Time Domain HRV

- Average heart rates in beats per minute = mean heart rates of each 5 min interval.
- SDNN in milliseconds = standard deviation of NN; this reflected the general variability of the heart rate influenced by the autonomic nervous system and also the endocrine and thermoregulatory mechanisms.
- pNN50 and pNN20 in percent = percent of NN intervals that differed more than 50/20 ms from the prior interval; this mainly reflected the parasympathetic influence.
- RMSSD in milliseconds = root mean square of the differences between successive NN intervals; this mainly reflected the parasympathetic influence.

#### 2.2.2. Stress Index

$\mathrm{Stressin}\;\mathrm{dex}=\frac{\mathrm{Amo}}{2\times Mo\times MxDMn}$

Where *Mo* is the modal value, the most common value of the RR intervals; *Amo* is the number of RR intervals corresponding with the mode as a percentage of the total number of all readings; and *MxDMn* is the variability width, the difference between the maximum and minimum RR intervals.

The stress index is becoming increasingly popular because it reacts sensitively to shifts in the vegetative balance between the sympathetic and parasympathetic nerves.

#### 2.2.3. Frequency Domain HRV

The Fourier transformation shows that HRV signals are concentrated into three different frequency bands:

- Very low frequency power (VLF = 0.00–0.04 Hz) in ms^2^: an uncertain physiological meaning.
- Low frequency power (LF = 0.04–0.15 Hz) in ms^2^: this is mediated by a sympathetic tone; however, the interpretation is controversial and may represent both sympathetic and parasympathetic activity.
- High frequency power (HF = 0.15–0.4 Hz) in ms^2^: “sinus arrhythmia” mediated by alternating levels of a parasympathetic tone.
- LF/HF ratio: often referred to as the balance between the sympathetic and parasympathetic tones.
- Total power (TP) in ms^2^: this measures the total variance in HRV.

### 2.3. Statistics

All analyses were performed using IBM SPSS Statistics software, (IBM Corp. IBM SPSS Statistics for Windows, Version 26.0, Armonk, NY, USA). For the descriptive statistics, the data were expressed as the mean ± standard deviation. The study population was divided into three diagnosis groups and one healthy control group. An unpaired *t*-test was used to compare the differences between the healthy control group and each patient group (Table 1). Significant group differences were anticipated if the *p*-value was <0.05. 

## 3. Results

**Figure 1:**
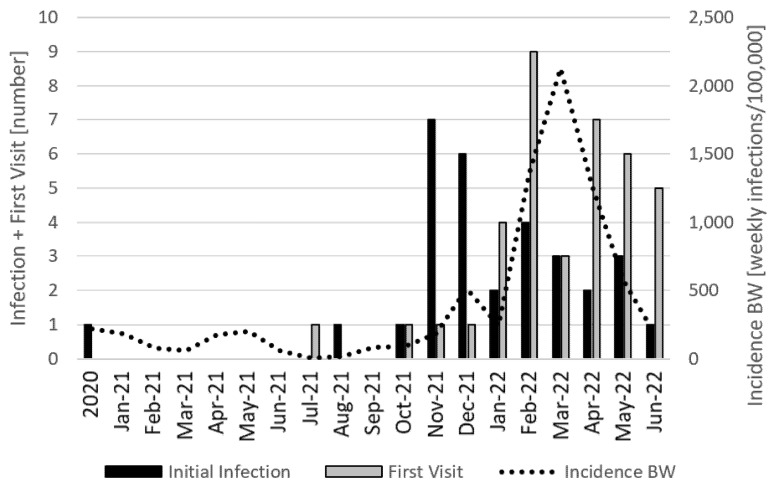
Timeline between initial SARS-CoV-2 infection/vaccination and first visit of cause of post-acute sequelae (PASC). **X-axis:** time; **left Y-axis**: number of initial infections and first visits in this month. **Right Y-axis**: Monthly new SARS-CoV-2 infections in Baden-Württemberg (State in southern Germany, BW).

The time line, as illustrated in the Figure 1, showed that the initial COVID-19 infection of our study group had a peak in November 2021 when the Delta variant of the SARS-CoV-2 virus was dominant in Baden Württemberg (BW), a state of origin of our patients in southern Germany. Despite the high increases in new infections due to the Omicron variant of the SARS-CoV-2 virus up to February 2022, the number of initial infections in our analysis decreased. The first visit for ongoing health problems of our patients after COVID-19 or vaccination had a peak in February 2022, two months after the initial infection peak. There were only a few infections and first visits prior to November 2021. This mostly depended on the low infection rates of children in Germany due to a hard school lockdown. 

The following results of our analysis are displayed in Table 1. 

The age was not significantly different between the patient groups and the healthy control group (healthy control: 14.2 ± 3.8 years; Group 1: 13.3 ± 2.9 years; Group 2: 17.1 ± 3.7 years; Group 3: 14.9 ± 1.5 years). There were a few young adults up to 21 years of age in all groups. The body weight and height were not significantly different between the groups. The systolic blood pressure was normal and not significantly different. However, the diastolic blood pressure was significantly enhanced in the three patient groups (Group 1: 67.2 ± 9.1 mm Hg *; Group 2: 75.4 ± 8.6 mm HG ***; Group 3: 71.0 ± 6.3 mm Hg *) compared with the age-matched healthy control group (61.7 ± 11.2 mm Hg). The myocardial function, measured by fractional shortening and the left ventricular performance index, were normal in all patients and not significantly different between the patient groups. 

The heart rate increase during the 5 min standing was significantly higher in all patient groups (Group 1: 25.3 ± 11.7 bpm **; Group 2: 26.2 ± 14.2 bpm **; Group 3: 29.6 ± 12.8 bpm **) compared with the healthy controls (16.2 ± 7.1 bpm). The average heart rates were enhanced whilst lying and standing in all patient groups. However, the heart rate increase depended on significantly higher heart rates whilst standing (Group 1: 106.0 ± 13.1 bpm ***; Group 2: 102.9 ± 10.8 bpm ***; Group 3: 115.1 ± 12.8 bpm ***) compared with 89.8 ± 13.2 bpm in the healthy controls. The stress index was enhanced whilst lying in all patient groups, but was more pronounced whilst standing (Group 1: 343.0 ± 292.0 ***; Group 2: 319.2 ± 219.3 **; Group 3: 494.1 ± 340.8 ***) compared with the healthy controls (168.5 ± 116.5). Our HRV data indicated that the heart rate increase whilst standing in children with dysautonomia after COVID-19 depended on a collapse of the vagus, with significantly lower pNN20 values (Group 1: 27.5 ± 17.4% ***; Group 2: 20.6 ± 13.5% ***; Group 3: 15.6 ± 13.6% ***) compared with the healthy control group (78.6 ± 10.8%). However, the RMSSD as well as the high frequency power data, which also depend on the vagus activity, were not clearly different in the patient groups compared with the healthy controls. This uncertainty depended on the high standard deviations of the frequency domain data. The respiratory rates were significantly increased in patient Groups 1 and 2 (healthy control: 16.3 ± 3.3/min; Group 1: 18.8 ± 2.1/min **; Group 2: 19.1 ± 4.0/min *; Group 3: 17.7 ± 1.8/min). The quality of measurement, shown as analysable heart beats per measurement, was not significantly different between the groups (healthy control: 82.7 ± 18.5%; Group 1: 82.5 ± 15.3%; Group 2: 80.8 ± 24.7%; Group 3: 79.1 ± 17.0%).

## 4. Discussion

Persistent health problems after COVID-19 and longer than 11 weeks on average in our patient group of children and young adults up to 21 years of age overlapped with many symptoms that were difficult to specify. What they all had in common was a deterioration in physical and/or mental performance, with a significant impact on school performance. These problems were well-known prior to the COVID-19 pandemic, but have now increased further; perhaps due to the disease, but probably also as a consequence of health policy measures to prevent the spread of the pandemic such as lockdowns or vaccinations. As the perception of the burden of illness of our patients was influenced by a controversial public discussion about these political interventions, we looked for methods that could make an objective assessment possible. 

The analysis of heart rate variability is an established method to calculate work performance [9]. Based upon our experience with the HRV analysis in children, we have used HRV monitoring since the beginning of the pandemic; thus, we were able to describe in detail the effects of the SARS-CoV-2 infection on the autonomic nervous system. We now present the first analysis of HRV data in children with long-term health problems after COVID-19 and its vaccination. 

The results showed comparable highly significant effects on the heart rate whilst lying and standing in all patient groups who shared the fact that their immune system had been confronted with the spike protein of the SARS-CoV-2 virus, either through the infection and/or the vaccination. In summary, exactly half of our patient group had two mRNA vaccinations on average, which agreed well with the current vaccination rate of 67% for this age group in Baden-Württemberg, Germany. 

Interestingly, the elevated heart rate in our children proceeded with elevated diastolic blood pressure, as recently shown in adults with PASC [10]. A subclinical diastolic impairment without a systolic involvement may be observed in patients with COVID-19, measured with the myocardial performance index [11]. However, our data clearly indicated a complete myocardial recovery in all our patients.

Together with many publications about dysautonomia in adults with PASC [12], there seems to be no question that the clinical symptoms of PASC are an expression of dysautonomia with elevated heart rates; however, the underlying pathophysiology seems to be unclear. The interpretation of the HRV data with elevated heart rates together with low pNN20/50 values may indicate a vagus weakness [13]. However, our RMSSD data and the frequency domain analysis were difficult to interpret due, in part, to the high standard deviations. 

It has been shown that autoantibodies against G-coupled receptors have an impact on HRV and may cause dysautonomia. These autoantibodies are elevated in adults with PASC [14]. It seems plausible that autoimmunity has an impact on the heart rate and blood pressure independent of the sympatico vagal balance. 

Therapeutically, it seems necessary to decrease elevated heart rates primarily to improve the clinical symptoms. However, eight patients fulfilled the criteria of postural orthostatic tachycardia syndrome and nine patients of an inappropriate sinus tachycardia, who were treated according the current guidelines [8]. Many other patients had heart rates just below our limits of a heart rate increase with average heart rates whilst lying and standing of more than 35 bpm or a lying heart rate of more than 90 bpm. Three athletes had low heart rates whilst lying but suffered from a heart rate increase whilst standing, as recently published in a case report [15].

In addition to a clinical improvement, there is increased evidence to treat elevated resting heart rates that are related to cardiovascular disease, cancer, and all-cause mortality [16]. This prognostic impact was even shown in adolescents by Swedish register data [17]. In the first step, we advise to increase sports [18] and supplement with omega-3 fatty acids, which significantly reduce the heart rate [19] and cardiovascular mortality [20]. Many of our patients required pharmacotherapy with low-dose propranolol, ivabradine, or midodrine; several of the children had to take this pharmacotherapy for up to one year after several unsuccessful attempts to exit.

It is not possible to make any recommendations for the SARS-CoV-2 vaccination based upon our data. However, vaccinations do not safely protect against PASC. Current publications suffer from significant methodological limitations such as a vaccination rate of only 1.6% in one publication of big data [3]. The apparently lower incidence of PASC after a natural infection with the Omicron variant could be an indication that a mucosal immunity is better protection against PASC than the systemic immunity of the current vaccinations. A number of such intranasal vaccines against SARS-CoV-2 are being tested in clinical trials [21]. 

## 5. Limitations

This retrospective analysis had a few limitations. First of all, the data of the healthy control grouped were measured prior to the pandemic. We are aware that the lockdown had an effect on heart rate variability, as recently published [22]. We used our published healthy control group and proved the matching according to age, height, weight, and gender. Patients with somatic and psychosomatic problems were carefully excluded.

## Figures and Tables

**Table 1:** Basic data and HRV analysis of the active standing test.

	Healthy Control	Post-COVID-19	Post-COVID-19 + VAC	Post-VAC
**Patients**	47	19	12	7
**Gender (female/male)**	30/17	13/6	8/4	4/3
**Age (years)**	14.2 ± 3.8	13.3 ± 2.9	17.1 ± 3.7	14.9 ± 1.5
**Height (cm)**	160.1 ± 14.2	154.8 ± 10.9	166.4 ± 10.3	161.9 ± 5.5
**Weight (kg)**	52.6 ± 14.3	47.9 ± 13.1	60.1 ± 11.3	49.7 ± 7.2
**Systolic BP (mmHg)**	114.5 ± 9.2	115.4 ± 13.7	119.1 ± 9.7	120.7 ± 9.7
**Diastolic BP (mmHg)**	61.7 ± 11.2	67.2 ± 9.1 *	75.4 ± 8.6 ***	71.0 ± 6.3 *
**Fractional shortening (%)**	>30	37.4 ± 4.1	40.6 ± 3.7	35.40 ± 1.80
**Myocardial Performance Index**	<0.4	0.14 ± 0.06	0.13 ± 0.10	0.18 ± 0.13
**Heart Rate Increase (bpm)**	16.2 ± 7.1	25.3 ± 11.7 **	26.2 ± 14.2 **	29.6 ± 12.8 **
**Lying**				
**Mean Heart Rate (bpm)**	71.9 ± 8.1	80.7 ± 15.9 *	76.7 ± 16.5	85.4 ± 15.4 **
**Stress Index (pt)**	97.8 ± 85.1	150.7 ± 202.7 *	183.6 ± 140.4 ***	201.7 ± 213.6 **
**SDNN (ms)**	89.1 ± 38.4	68.7 ± 26.4 *	71.8 ± 64.7	75.3 ± 50.4
**pNN20 (%)**	78.6 ± 10.8	68.8 ± 26.6	56.0 ± 28.5 ***	53.3 ± 32.9 ***
**RMSSD (ms)**	85.1 ± 56.2	68.7 ± 38.5	77.2 ± 94.6	64.7 ± 66.8
**Power HF Band (ms^2^)**	2920 ± 2229	2097 ± 2442	2376 ± 5298	3301 ± 6482
**Power LF Band (ms^2^)**	1518 ± 2795	794 ± 774	1132 ± 1800	788 ± 674
**Power VLF Band (ms^2^)**	1553 ± 2182	1099 ± 764	2612 ± 4472	1907 ± 2385
**Power Total (ms^2^)**	5819 ± 6203	3990 ± 3260	6119 ± 11,289	5997 ± 7248
**LF/HF Ratio**	0.97 ± 1.1	0.98 ± 1.23	1.48 ± 1.52	1.61 ± 1.69
**Standing**				
**Mean Heart Rate (bpm)**	89.8 ± 13.2	106.0 ± 13.1 ***	102.9 ± 10.8 ***	115.1 ± 12.8 ***
**SDNN (ms)**	58.0 ± 22.7	43.9 ± 19.4 **	62.9 ± 48.8	38.8 ± 16.1
**pNN20 (%)**	45.8 ± 18.6	27.5 ± 17.4 ***	20.6 ± 13.5 ***	15.6 ± 13.6 ***
**RMSSD (ms)**	40.4 ± 22.7	26.7 ± 22.2 *	30.9 ± 48.9	22.4 ± 22.3
**Stress Index (pt)**	168 ± 116	343 ± 292 ***	319 ± 219 **	494 ± 340 ***
**Power HF Band (ms^2^)**	949 ± 1222	939 ± 2334	642 ± 1905	1184 ± 2640
**Power LF Band (ms^2^)**	1331 ± 1115	1623 ± 5006	785 ± 762	1276 ± 1725
**Power VLF Band (ms^2^)**	1299 ± 1506	1058 ± 2197	1309 ± 1926	948 ± 848
**Power Total (ms^2^)**	3579 ± 3012	3611 ± 9437	2737 ± 4144	3409 ± 4895
**LF/HF Ratio**	2.54 ± 1.95	3.13 ± 2.96	9.41 ± 9.63	4.20 ± 3.50 *
**Respiratory Rate (1/min)**	16.3 ± 3.3	18.8 ± 2.1 **	19.1 ± 4.0 *	17.7 ± 1.8
**Number of Beats**	792 ± 111	902 ± 139 **	883 ± 168 *	895 ± 192 *
**Quality (%)**	82.7 ± 18.5	82.5 ± 15.3	80.8 ± 24.7	79.1 ± 17.0

The *t*-test between healthy control group and patient groups: * *p*-value < 0.05; ** *p*-value < 0.01; *** *p*-value < 0.001.

## Data Availability

Anonymized data are available from the author.

## Abbreviations

BW: Baden-Württemberg; HF: high frequency power; HF/LF: ratio of HF to LF; HRV: heart rate variability; LF: low frequency power; PASC: post-acute sequalae of SARS-CoV-2 infection; pNN20: percent of NN intervals that differed more than 20 ms from the prior interval; SDNN: standard deviation of all NN intervals; RMSSD: the square root of the mean of the sum of the squares of the differences between the adjacent NN intervals; VLF: very low frequency power.
